# QuASeR: Quantum Accelerated de novo DNA sequence reconstruction

**DOI:** 10.1371/journal.pone.0249850

**Published:** 2021-04-12

**Authors:** Aritra Sarkar, Zaid Al-Ars, Koen Bertels

**Affiliations:** 1 Department of Quantum and Computer Engineering, Faculty of Electrical Engineering, Mathematics and Computer Science, Delft University of Technology, Delft, The Netherlands; 2 Department of Informatics Engineering, Faculty of Engineering, University of Porto, Porto, Portugal; University of Massachusetts Lowell, UNITED STATES

## Abstract

In this article, we present QuASeR, a reference-free DNA sequence reconstruction implementation via de novo assembly on both gate-based and quantum annealing platforms. This is the first time this important application in bioinformatics is modeled using quantum computation. Each one of the four steps of the implementation (TSP, QUBO, Hamiltonians and QAOA) is explained with a proof-of-concept example to target both the genomics research community and quantum application developers in a self-contained manner. The implementation and results on executing the algorithm from a set of DNA reads to a reconstructed sequence, on a gate-based quantum simulator, the D-Wave quantum annealing simulator and hardware are detailed. We also highlight the limitations of current classical simulation and available quantum hardware systems. The implementation is open-source and can be found on https://github.com/QE-Lab/QuASeR.

## Introduction

Understanding the genome of an organism reveals insights [1] with scientific and clinical significance like causes that drive cancer progression, intra-genomic processes influencing evolution, enhancing food quality and quantity from plants and animals. Genomics data is projected to become the largest producer of big data within the decade [2], eclipsing all other sources of information generation, including astronomical as well as social data. At the same time, genomics is expected to become an integral part of our daily life, providing insight and control over many of the processes taking place within our bodies and in our environment. An exciting prospect is personalized medicine [3], in which accurate diagnostics can identify patients who can benefit from precisely targeted therapies. Despite the continual development of tools to process genomic data, current approaches are yet to meet the requirements for large-scale clinical genomics. In this case, patient turnaround time, ease-of-use, robustness and running costs are critical. As the cost of whole-genome sequencing (WGS) continues to drop [4], more and more data is churned out creating a staggering computational demand. Therefore, efficient and cost-effective computational solutions are necessary to allow society to benefit from the potential positive impact of genomics. This paper provides more efficient solutions to the high computational demands in the field of genomics.

**Fig 1:**
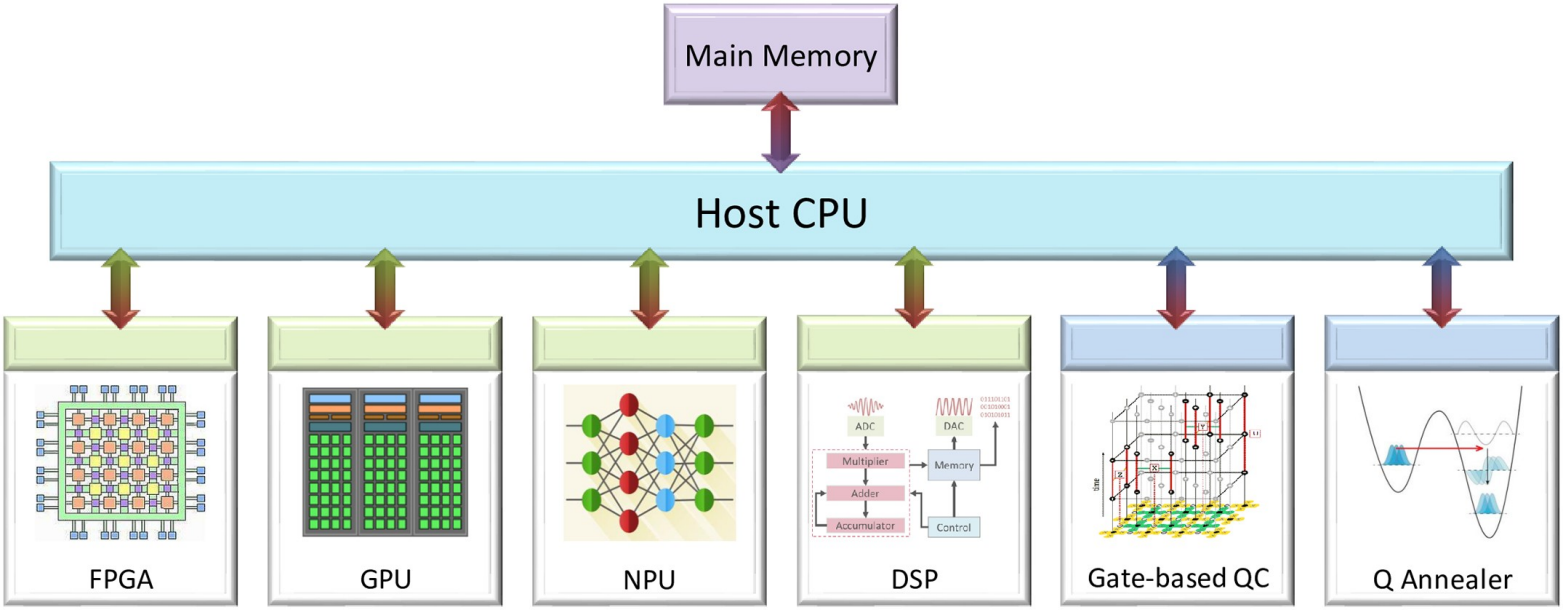
The accelerator model of computing.

The last decade in computer architecture has focused on the emergence of accelerators [5] as specialized processing units to which the host processor offloads suitable computational tasks. This is motivated by the various technological bottleneck (such as power use, frequency, memory access, level of parallelism) to increasing the computational capabilities of generic CPUs. Different accelerators, as shown in Fig 1, are chosen based on the their strengths that enable better execution of a particular type of logical manipulation. Commonly used accelerators today include field-programmable gate arrays (FPGA), graphics-processing units (GPU), neural processing units (NPU), digital signal processors (DSP), etc.

An alternate computing paradigm that is receiving a lot of attention lately in computer architecture is quantum computing—which uses fundamental properties of quantum mechanics to achieve a computational advantage over classical computation. Within the next decade, it is likely that applications will be a hybrid combination of a classical computer and a quantum accelerator, with multiple computational kernels, than a universal quantum processing unit. As a holistic view we consider two classes of quantum accelerator [6] as additional co-processors. One is based on quantum gates and the second is based on quantum annealing. The classical host processor keeps the control over the total system and delegates the execution of certain parts to the available accelerators.

In this paper, we propose an implementation of a DNA sequence reconstruction technique (called de novo assembly) on a quantum computing platform. De novo assembly using the overlap-layout-consensus method has many advantages over other simpler methods, but suffers from large computational complexity, which motivates targeting a quantum accelerator. We target both a gate-based quantum system as well as a quantum annealer. Each step of the formulation is explained with simple examples [7] to target both the genomics research community and quantum application developers. The implementation is evaluated on the D-Wave simulator, the D-Wave annealer in the cloud and the QX Simulator. The current limitations in solving real problem sizes to achieve a quantum advantage are discussed. It is the first time this important computational problem of de novo assembly in bioinformatics is targeted on a quantum accelerator with the full description of the pipeline.

Recently [8], also discussed de novo sequencing on quantum annealing and quantum-inspired annealing, citing a preprint of our research as presented in this paper. We appreciate this research done independently to ours echoing a similar motivation to explore the applicability of quantum computing to DNA sequence reconstruction and reaching similar results. In contrast to their work, we additionally target the gate based quantum computing model, which is considered to be the future standard of quantum acceleration. Furthermore, this paper is presented from the perspective of a quantum application developer, with details from genomics, quantum and computer science formulated in a self-contained matter. In addition, an example is implemented via the various steps of the algorithm and the execution results are demonstrated as a proof-of-concept to evaluate the quality, scalability and limitations.

This paper is organized as follows. In the section “DNA sequence reconstruction”, we introduce the specific problem of sequence reconstruction via de novo assembly using the overlap-layout-consensus approach. This is the target algorithm for which the quantum kernel is formulated. The section “Quantum accelerated optimization” first introduces the required technical background for the tools for formulating the de novo assembly problem, i.e. quantum accelerator, QUBO, TSP, Hamiltonians and the gate-based QAOA variational hybrid approach. In the section “Implementing de novo assembly”, the de novo assembly problem is mapped to a TSP and then the TSP is mapped to a QUBO. A small proof-of-concept example is detailed at each step. The section “Solving on a quantum system” systematically solves the QUBO formulation first on the D-Wave simulator and annealer system, and thereafter a QAOA on OpenQL is attempted. Then we discuss the limitations for scaling the formulation on real datasets using classical simulation and available quantum hardware systems. The “Conclusion” section concludes the paper.

## DNA sequence reconstruction

In order to sequence the DNA of an organism, DNA is broken down into fragments since DNA sequencing machines are not capable of reading the entire genome at once. Then these fragments are sequenced using modern sequencing technologies (such as Illumina), which produces reads of approximately 50-150 base pairs at a time, with some known error rate. To reconstruct the DNA from these fragments for further analysis, two different techniques are used (a) ab initio reference-based alignment of reads and (b) de novo reference-free assembly of reads. In our past research [9], we had implemented a quantum variants of reference-based alignment, while in this article, we focus on de novo assembly. Since the principles of quantum computation are fundamentally different, we will investigate the most basic algorithmic primitive for which the quantum kernel can be constructed. Thus, before presenting the corresponding quantum algorithm, the existing classical algorithms are reviewed here.

### De novo reference-free assembly

During sequencing, multiple copies of the DNA are made before fragmenting it. Thus, a portion of the data is preserved in multiple copies which are chopped off at different places resulting in data overlaps which facilitate stitching. This method is called de novo assembly, as no other data than the sequenced read is used for reconstruction. It is computationally expensive and done normally for the first time a new species is sequenced.

There are different methods [10] for de novo assembly used by the available tools: Overlap-Layout-Consensus (OLC) methods, de Bruijn graph (DBG) methods, string graphs, greedy and hybrid algorithm, etc. Real-world WGS data induces various problems in all these methods. Examples are spurs (short, dead-end divergences from the main path), bubbles (paths that diverge then converge), frayed rope pattern (paths that converge then diverge) and cycles (paths that converge on themselves) [11]. Common causes of these complexities are attributed to repeats in the target and sequencing error in the reads. Most optimal graph reductions belong to the NP-hard class of problems, thus assemblers (like Euler, Velvet, ABySS, AllPaths, SOAPdenovo) rely on heuristics to remove redundancy, repair errors or otherwise simplify the graph. The choice of algorithms is based on the quality, quantity and structure of the genome data. Current short-read sequencing technologies produce very large numbers of reads favoring DBG methods. However, single molecule sequencing from third generation sequencing machines produces high-quality long reads, which could favor OLC methods.

In DBG, the nodes represent all possible fixed-length strings of length K (K-mer graph). The edges represent fixed-length suffix-to-prefix perfect overlaps between sub-sequences that were consecutive in the larger sequence. In WGS assembly, the K-mer graph represents the input reads. Each read induces a path and those with perfect overlaps induce a common path as an advantage, however, compared to overlap graphs, K-mer graphs are more sensitive to repeats and sequencing errors as K is much less than read size. In an ideal construction, the Eulerian path corresponds to the original sequence, though graphs built from real sequencing data are more complicated.

**Fig 2:**
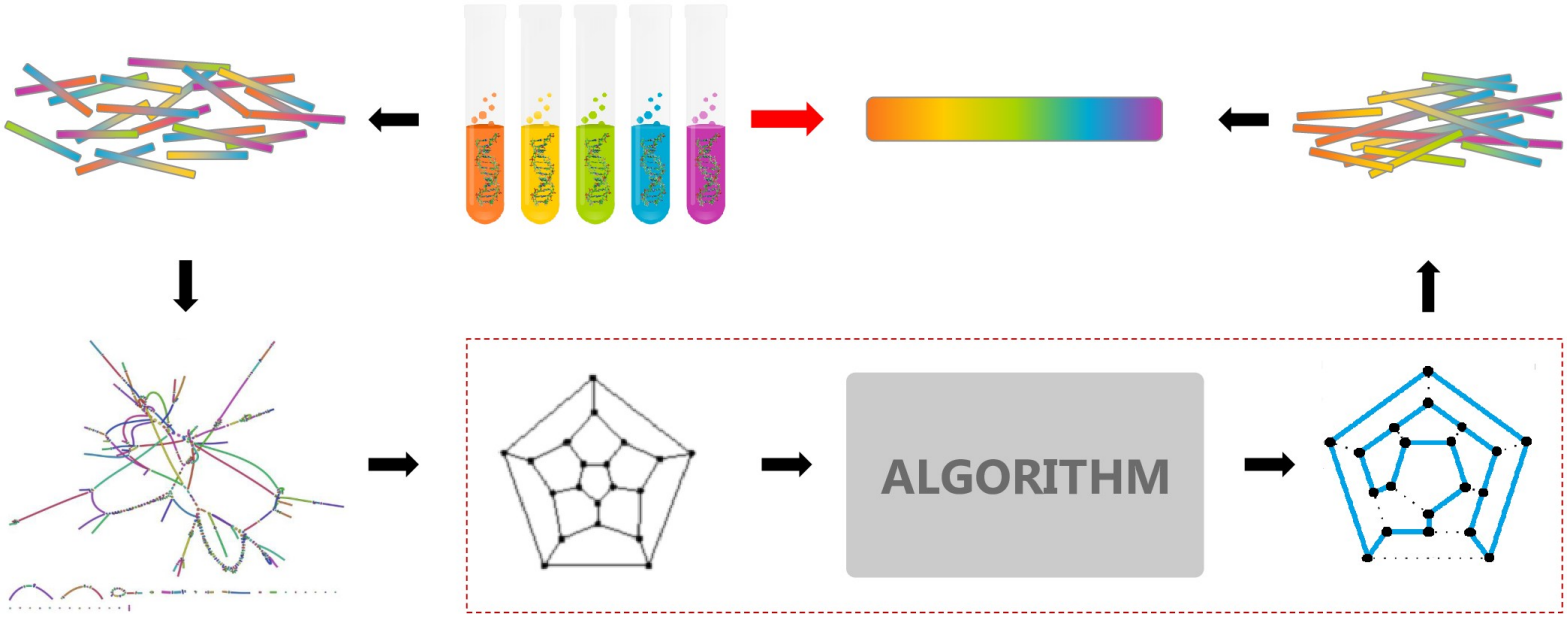
Overlap-layout-consensus genome assembly algorithm.

In the OLC method [12], as shown in Fig 2, an overlap graph represents the sequencing reads as nodes and their overlaps (pre-computed by pair-wise sequence alignments) as edges. Paths through the graph are the potential assembled DNA pieces and can be converted to sequence. Formally, this represents a Hamiltonian cycle, a path that travels to every node of the graph exactly once and ends at the starting node, including each read once in the assembly. There is no known efficient algorithm for finding a Hamiltonian cycle as it is in the NP-complete class. Though it was feasible for microbial genome (in 1995) and the human genome (in 2001), NGS projects have abandoned it due to the high computational burden to be commercially viable. This is the target for quantum acceleration in this research.

## Quantum accelerated optimization

The OLC method of de novo assembly requires solving the minimum Hamiltonian cycle problem. This is also famously known as the Traveling Salesman Problem (TSP), which belongs to the NP-hard class of computational complexity. Thus, we need to formulate it as an approximate optimization problem as even quantum computers cannot solve NP-hard problems in polynomial time.

**Fig 3:**
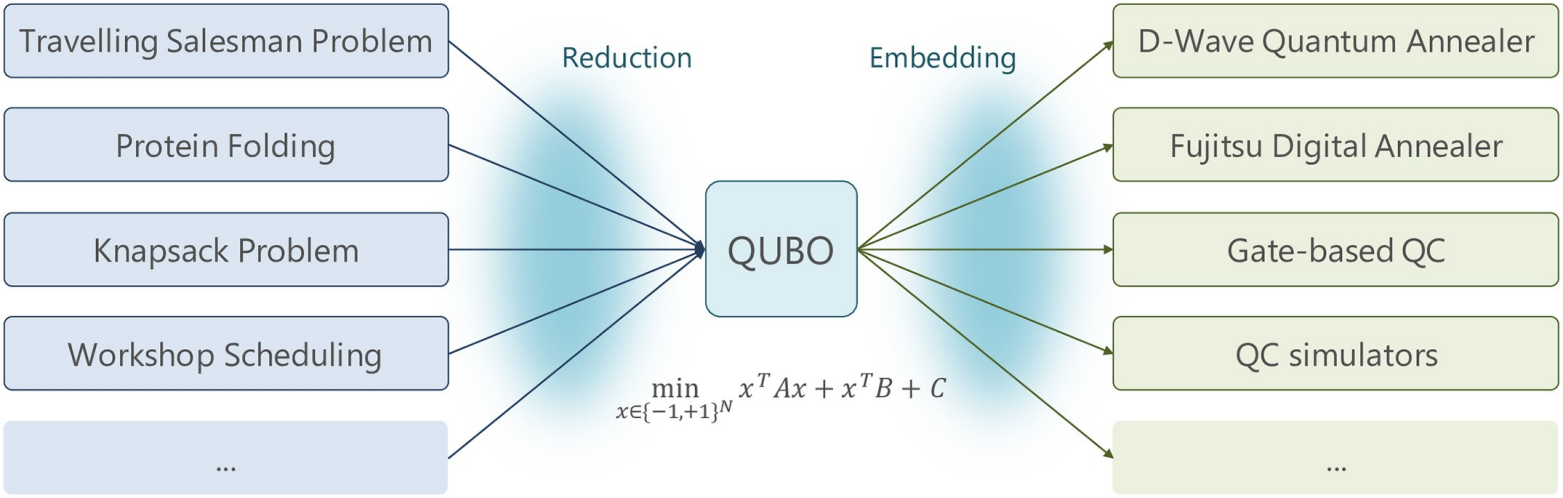
QUBO reduction from NP-hard problems.

Mapping an NP-hard problem to quantum involves 2 steps as shown in Fig 3. The first step is to *reduce* the given application to a Quadratic Unconstrained Binary Optimization (QUBO). The second step is to *embed* the QUBO to the connectivity structure of the hardware.

In this section, we first introduce the two models of quantum computation that we focus on: the gate-based circuit model and quantum annealing. Thereafter, we briefly review the formulation of a classical QUBO and TSP. Finally, we present the Hamiltonian formulation in adiabatic quantum computing, for optimization problems in quantum annealing and gate-based variational hybrid approach.

### Quantum accelerator models

There are several models of quantum computation. The theoretical models, like the quantum circuit model, adiabatic quantum computing, measurement-based (cluster state) quantum computation and topological quantum computing are equivalent to each other within polynomial time reduction.

One of the most popular and by far the most extensively developed is the circuit model for gate-based quantum computation [13]. This is the conceptual generalization of Boolean logic gates (e.g. AND, OR, NOT, NAND, etc.) used for classical computation. The gate set for the quantum counterpart allows a richer diversity of states on the complex vector space (Hilbert space) formed by qubit registers. The quantum gates, by their unitary property, preserve the 2-norm of the amplitude of the states thereby undergoing a deterministic transformation of the probability distribution over bit strings. The power of quantum computation stems from this exponential state space evolving in superposition while interacting by interference of the amplitudes. Gate-based quantum algorithms are designed such that the solution states interfere constructively while the non-solutions interfere destructively, biasing the final probability distribution in favor of reading out the solution(s).

Another common type of a quantum system is a quantum annealer. Quantum annealing is connected to the adiabatic quantum computing paradigm [14], although there are subtle differences. While the circuit model was inspired by Boolean logic circuits, quantum annealing was inspired by the metallurgical process of annealing where by virtue of thermal fluctuations, the material is able to explore more favorable parameters (e.g. crystal size) by thermally jumping over barriers in the parameter space. By imparting heat (energy), the solution parameters (like a ball) can climb a local minima (like a mountain and discover a deeper valley on the other side). Quantum annealing uses quantum fluctuations instead to tunnel through high but thin barriers in the target function. If the parameter landscape have these specific kind of barriers it can translate to a computational speedup in finding the minimum (ground state) of the function.

The technique to formulate a problem and program the corresponding computing model is considerably different. This is presented in the next sections.

### Quadratic unconstrained binary optimization

A binary quadratic model (BQM) comprises a collection of binary-valued variables that can be assigned two chosen values (based on the model) with associated linear and quadratic biases. Two isomorphic BQM are:

- QUBO models: *x*_*i*_ ∈ {0, 1} Boolean values
  $$\begin{array}{c} H\left(x\right)=\sum_{i}Q_{ii}x_{i}+\sum_{i,j}Q_{ij}x_{i}x_{j}+k \end{array}$$
- Ising models: *σ*_*i*_ ∈ {−1, + 1} spin states
  $$\begin{array}{c} H\left(\sigma \right)=-\mu \sum_{i}h_{i}\sigma_{i}-\sum_{i,j}J_{ij}\sigma_{i}\sigma_{j} \end{array}$$

The choice of model depends on the problem. Using QUBO, it might be easier to write numbers in standard binary notation (e.g. 101_2_ = 5_10_) in the optimization problem; or it might be required to destructively interfere two variable (e.g. two battling Pokemons) using the Ising model. Quantum processors like annealers use the Ising model, thus QUBO equations need to be converted into Ising under the hood.

QUBO model [15, 16] unifies a rich variety of combinatorial optimization problems as an alternative to traditional modeling and solution methodologies. These problems are concerned with making wise choices in settings where a large number of yes/no decisions must be made and each set of decisions yields a corresponding objective function value—like a cost or profit value. The QUBO model is expressed by the optimization problem:

$$\begin{array}{c} \text{minimize}\;y=x^{t}Qx=H\left(x\right) \end{array}$$

where *x* is a vector of binary decision variables and *Q* is a (symmetric or in upper triangular) square matrix of constants.

Different types of constraining relationships arising in practice can be embodied within the unconstrained QUBO formulation using penalty functions. The penalties introduced are chosen so that the influence of the original constraints on the solution process can alternatively be achieved by the natural functioning of the optimizer as it looks for solutions that avoid incurring the penalties. Penalties are not unique, meaning that many different values can be successfully employed. For a particular problem, a workable value is typically set, based on domain knowledge and on what needs to be accomplished. If a constraint must be satisfied, i.e., a “hard” constraint, then the penalty must be large enough to preclude a violation. More moderate penalties are set for “soft” constraints, meaning that it is desirable to satisfy them but slight violations can be tolerated. Casting the QUBO model as a minimization problem permits a maximization problem to be solved by minimizing the negative of its objective function.

QUBO models belong to the NP-hard class of problems. Thus exact solvers (e.g. CPLEX, Gurobi) work practically only for very small problem instances (around 100 variables) using mostly branch-and-bound or problem-specific techniques. However, impressive successes are being achieved by using meta-heuristic methods that are designed to find high quality but not necessarily optimal solutions in a modest amount of computer time. Among the best meta-heuristic methods for QUBO are those based on tabu search, path relinking, simulated annealing, genetic/memetic strategies, and their ensembles. Recently, with the availability of small-scale quantum processors, there is a huge research thrust in achieving quantum advantage for QUBO models.

### Traveling salesman problem

A Hamiltonian path is a graph path between two vertices of a graph that visits each vertex exactly once. If a Hamiltonian path exists whose endpoints are adjacent, then the resulting graph cycle is called a Hamiltonian cycle. It is a path that starts from one node and ends at the same node covering all the nodes of that graph.

Given a directed complete graph *G* = (*V*, *E*) with weights *w*_*ij*_ on the directed edge *i* → *j*, the directed traveling salesman problem (TSP) aims to find a directed Hamiltonian cycle of minimum weight, i.e., a cycle that visits all nodes (cities) of the graph and such that the sum of the edge weights (travel cost) is minimum. Intuitively, given the ordered pair-wise distance between cities, the TSP involves finding the shortest route that visits every city once. The order of visiting the cities are not constrained.

TSP falls under the NP-hard class (thus outside BQP), so the time to find the exact solution scales exponentially also on a quantum computer with the problem size. Often a good sub-optimal solution is admissible, thus heuristic algorithms of much lesser complexity can be employed. TSP solvers are used in many industrial applications in the domains of planning, scheduling, logistics, packing, DNA sequencing, network protocols, telescope control, VLSI testing, and many more.

The first step to specifying a TSP is to create a (weighted) graph specifying the edges in the format (vertex-from; vertex-to; weight). Next, the TSP graph is transformed into a QUBO graph. QUBO variables are labeled (*n*, *t*) where *n* is a node (read) in the TSP graph and *t* is the time index of visiting it in order. E.g., if (*a*, 0) = 1 in the solution state, that means the node *a* is visited first. Since the total number of visits (time IDs) equals the total number of nodes (read IDs); the total possible combinations of (*n*, *t*) is |*G*|^2^. |*G*| is the number of nodes in the original TSP graph.

The QUBO graph will have 2 * |*G*|^2^ * (|*G*| − 1) interactions (or edges). The interactions denote pairs of 2 nodes that can/cannot coexist. The weight of the interaction shows the reward/penalty of coexisting. A higher positive value denotes more penalty. There are 3 types of penalty, for multi-location (being at 2 places at the same time), repetition (being at a city twice) and path cost for the tour.

### Hamiltonian formulation

In physical systems (classical or quantum), a Hamiltonian describes the energy of an object. More specifically, it describes the time-evolution of a system expressed by the Schrödinger equation:

$$\begin{array}{c} i\hbar \frac{d}{dt}\left|\psi \left(t\right)\right\rangle =H|\psi \left(t\right)\rangle \end{array}$$

The unitary operator underlying the Hamiltonian is obtained by solving the equation for some time duration:*U* = exp(−*iHt*/ℏ). The time-independent formulation of the equation reflects the total energy of the system *E* = *H*|*ψ*〉.

The adiabatic theorem dictates that if the change in the time-dependent Hamiltonian occurs slowly, the resulting dynamics remain simple, i.e. starting close to an eigenstate, the system remains close to an eigenstate. For a quantum mechanical system, some initial Hamiltonian *H*_*i*_ is slowly changed to some other final Hamiltonian *H*_*f*_. This implies that, if the system is started in the ground state (lowest eigenstate) of the initial Hamiltonian, the system will evolve to the ground state of the final configuration. The computational advantage comes from the choice of an easy-to-prepare quantum system like:

$$\begin{array}{c} H_{i}=-\sum_{i}\sigma_{i}^{X} \end{array}$$

(the ground state is the equal superposition state) and evolve the Hamiltonian to a system such that the ground state encodes the solution of the optimization problem we are interested in.

The change needs to be carried out by a defined schedule, for example, linear in the time scale *t* ∈ [0, 1] defined as:

$$\begin{array}{c} H\left(t\right)=\left(1-t\right)H_{i}+tH_{f} \end{array}$$

The energy difference between the ground state and the first excited state is called the gap, Δ(*t*). If *H*(*t*) has a finite gap for each *t* during the transition the system can be evolved adiabatically with the evolution speed proportional to 1/min(Δ(*t*))^2^. The gap, however, is highly problem-dependent, tending to have an exponentially small gap for hard problems (like those in the NP-hard class), making the time exponentially long. Thus it is unlikely that an exact solution for these problems can be found in polynomial time.

In adiabatic quantum computations, universal calculations are performed by mapping the problem to a final Hamiltonian defined as:

$$\begin{array}{c} H_{f}=-\sum_{<i,j>}J_{ij}\sigma_{i}^{Z}\sigma_{j}^{Z}-\sum_{i}h_{i}\sigma_{i}^{Z}-\sum_{<i,j>}g_{ij}\sigma_{i}^{X}\sigma_{j}^{X} \end{array}$$

Thus the system Hamiltonian *H*(*t*) becomes:

$$\begin{array}{c} H\left(t\right)=\left(1-t\right)[-\sum_{i}\sigma_{i}^{X}]+t[-\sum_{<i,j>}J_{ij}\sigma_{i}^{Z}\sigma_{j}^{Z}-\sum_{i}h_{i}\sigma_{i}^{Z}-\sum_{<i,j>}g_{ij}\sigma_{i}^{X}\sigma_{j}^{X}] \end{array}$$

The values of biases and couplings are set by the user/programmer for a quantum annealer.

The major drawback to implementing an adiabatic quantum computing directly is calculating the speed limit, which is harder than solving the original problem of finding the ground state of a Hamiltonian. Quantum annealing [17] drops the strict requirements of respecting speed limits in favor of repeating the transition multiple times. Sampling from the solutions is likely to find the lowest energy state of the final Hamiltonian (though there is no theoretical guarantee). Going from ‘nearly correct’ to ‘correct’ is still NP in general if the original problem is in the NP class of complexity (the parameters for local optima aren’t necessarily going to be distributed anywhere near the global optima). However, annealing can be useful if a sub-optimal solution is acceptable for the application.

### Variational hybrid approach

Coherent quantum protocols have promising exponential speedups but assume a fault-tolerant quantum computing platform, with high quality of qubits, a large number of gates and circuit width. These popular algorithms like Shor’s factorization, HHL for matrix inversion, though primitives for many quantum algorithms are not of immediate practical relevance. Wrapping these protocols in state preparation (from classical data to quantum) and state tomography (from quantum probability amplitudes of final state to classical statistics) can overrule the entire speedup achieved by the protocol itself.

**Fig 4:**
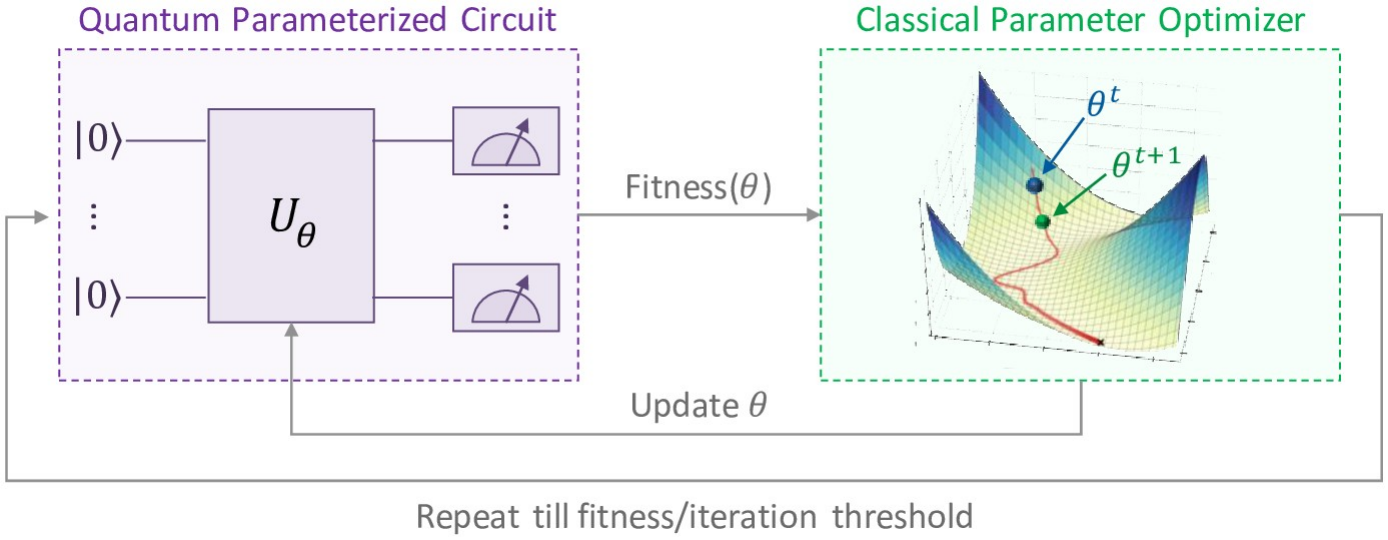
Variational hybrid quantum-classical approach for optimization.

Today, we are in the Noisy Intermediate-Scale Quantum (NISQ) era. These near-term quantum computers are more suited for hybrid quantum-classical (HQC) algorithms [18]. These are heuristic protocols based on the variational principle. In an HQC algorithm, all the power of the quantum computer is used for preparing a quantum state. The complexity of the algorithm is traded-off for multiple measurements over multiple cycles. The operations that require lots of gates on a quantum computer are offloaded to the classical computer (e.g. optimization, addition, division), which controls the quantum computer like an accelerator or a co-processor, as shown in Fig 4. A quantum circuit is defined as having a certain format *A* (or ansatz/stencil) with parameters. There are *m* parameters forming a parameter vector Λ_*m*_. These can be initialized randomly or with a classical guess. For the first cycle, the quantum computer takes the initial guess and evolves it using the circuit $A(\Lambda_{m}^{0})$. The Hamiltonian (energy) is measured out and sent to the classical computer. The variational principle updates the parameters in such a way that the energy of the Hamiltonian is lowered in each successive iteration. The optimization using $\Lambda_{m}^{1},\Lambda_{m}^{2},\Lambda_{m}^{3},\ldots$ continues until the acceptable threshold is satisfied, very similar to training in neural networks.

The variational principle forms the core theoretical basis behind the working of near-term quantum heuristic algorithms. It states that, for a trial wave-function (defining the family of quantum states reachable by varying the *m* parameters of *A*),

$$\begin{array}{c} E_{\Psi_{T}}=\frac{\langle \Psi_{T}\left(\Lambda \right){\hat{H}}_{QC}\Psi_{T}(\Lambda )\rangle }{\langle \Psi_{T}\left(\Lambda \right)|\Psi_{T}(\Lambda )\rangle }\geq E_{0} \end{array}$$

The normalization term 〈Ψ_*T*_(Λ)|Ψ_*T*_(Λ)〉 = 1 as we assume no leakage errors from the computational basis. Thus, it is possible to reach the ground-state energy by finding the right parameters. The more free the state is to represent quantum states (determined by the choice of *A*), the better it will be able to lower the energy.

Since adiabatic and gate systems offer effectively the same potential for achieving the gains inherent in quantum computing processes, analogous advances associated with QUBO models may ultimately be realized through quantum circuit systems as well.

An example of HQC algorithms is the Quantum Approximate Optimization Algorithm (QAOA) [19]. It is a hybrid variational algorithm that produces approximate solutions for combinatorial optimization problems. In theory, QAOA methods can be applied to more types of combinatorial optimization problems than embraced by the QUBO model [16]. The parameters of the QAOA framework must be modified to produce different algorithms to appropriately handle different problem types. QAOA is a polynomial-time HQC algorithm which can be seen as the Trotterization of an infinite time adiabatic algorithm. Since the AQC always gives the optimal solution for Hamiltonians with a non-zero gap, QAOA for infinite cycles also converges to the global optima.

The generalization of QAOA called the Quantum Alternating Operator Ansatz [20], consists of 2 Hamiltonians: a cost/problem Hamiltonian *H*_*C*_ (similar to the transverse field in AQC) and a driver/mixing Hamiltonian *H*_*M*_ (similar to the longitudinal field in AQC). This is repeated over *p* cycles with each Hamiltonian parameterized by the *γ* and *β* real values (rotation angles similar to the adiabatic evolution time). After this unitary evolution, the state is measured for the expectation value with respect to the ground state of the cost Hamiltonian. The initial state |*ψ*_0_〉 depends on the problem (typically either the all-zero or the equal superposition state).

$$\begin{array}{c} U\left(\theta \right)|\psi_{0}\rangle =H_{M}\left(\beta_{p}\right)H_{C}\left(\gamma_{p}\right)\ldots H_{M}\left(\beta_{2}\right)H_{C}\left(\gamma_{2}\right)H_{M}\left(\beta_{1}\right)H_{C}\left(\gamma_{1}\right)|\psi_{0}\rangle \end{array}$$

For an optimization instance, the user specifies the driver Hamiltonian ansatz, cost Hamiltonian ansatz, and the approximation order (cycles) of the algorithm. If the number of cycles in QAOA increases, theoretically, the sub-optimal solutions obtained can only get better, as the sub-optimal solutions defined by fewer cycles (with fewer free parameters) are always contained in more cycles (if the new rotation parameters are set to zero). However, practically, having more cycles causes difficulty for the classical optimizer to deal with more free parameters and can affect the convergence.

Since HQC trades off the decoherence issue of a long quantum circuit in the NISQ era with multiple low-depth, the number of repetitions required is high. To estimate the expectation of the prepared state in each optimization step, it needs to be (pre-rotated in the basis and) measured with respect to each Pauli term in the problem Hamiltonian and aggregated. Each Pauli term measurement in turn requires state tomographic trials.

Moreover, the optimizer might get stuck in local optima or barren plateaus [21] in the parameter landscape, requiring a few reruns to build confidence in the obtained optima. The HQC algorithms depend a lot on the choice of the classical optimizer as well. Here we experiment with the basic Nelder-Mead gradient-free optimizer, but many gradient-based and gradient-free choices exist (for example in libraries like SciPy in Python and TOMLAB in MATLAB) which needs to be chosen based on empirical testing of a particular formulation of the specific problem.

The pseudo-code for our OpenQL implementation of the QAOA algorithm is shown in Box 1.

**Box 1. Pseudo-code for OpenQL QAOA.**

• Invoke QAOA:

 ◊ Form parameterized cQasm [Input: reference circuit, ansatz, steps, coefficients, angle ids]

 ◊ Form parameters list [Input: gammas, betas]

 ◊ Set runtime deparameterized cQasm filename

 ◊ For each iteration:

  ⋆ For each Pauli product term in cost Hamiltonian (wsopp):

   ∘ Deparameterize cQasm [Input: parameterized QASM, parameter list]

   ∘ Add measurement basis rotation based on Pauli product term

   ∘ Invoke Qxelerator to aggregate measurement over shots

  ⋆ Calculate total Expectation value of trail state in cost Hamiltonian basis

  ⋆ Return value to optimizer. Save intermediate results via callback.

  ⋆ Classical optimizer updates parameter list

 ◊ Display cost function convergence, final parameters and optimized cost

## Implementing de novo assembly

In this section, first, we present a mapping from DNA to TSP, as common in the OLC method. Then the TSP is converted to a QUBO. A fully worked out example is presented as a proof-of-concept for quantum accelerated sequence reconstruction. The example is chosen based on the limit of currently available quantum computing hardware and classical simulators, however, the formulation and implementation is generic and scalable to industrial scale pipelines.

### DNA reads to TSP formulation

Suppose the sequencer produces reads of size 10 nucleotide bases and after removing duplicates, the reads obtained are:

read 0: ATGGCGTGCA

read 1: GCGTGCAATG

read 2: TGCAATGGCG

read 3: AATGGCGTGC

The pairwise overlap is calculated for each ordered pair based on how many prefix characters of the second string match exactly with the suffix of the first string. The edge weight is set to the negation of the overlap, as the constraints need to be formulated such that the path that *minimizes the overlap* is found.

The edge weights are {*(0,1):-7, (1,2):-7, (2,3):-7, (3,0):-9, (1,0):-3, (2,1):-3, (3,2):-3, (0,3):-1, (0,2):-4, (1,3):-4, (2,0):-6, (3,1):-6*}

**Fig 5:**
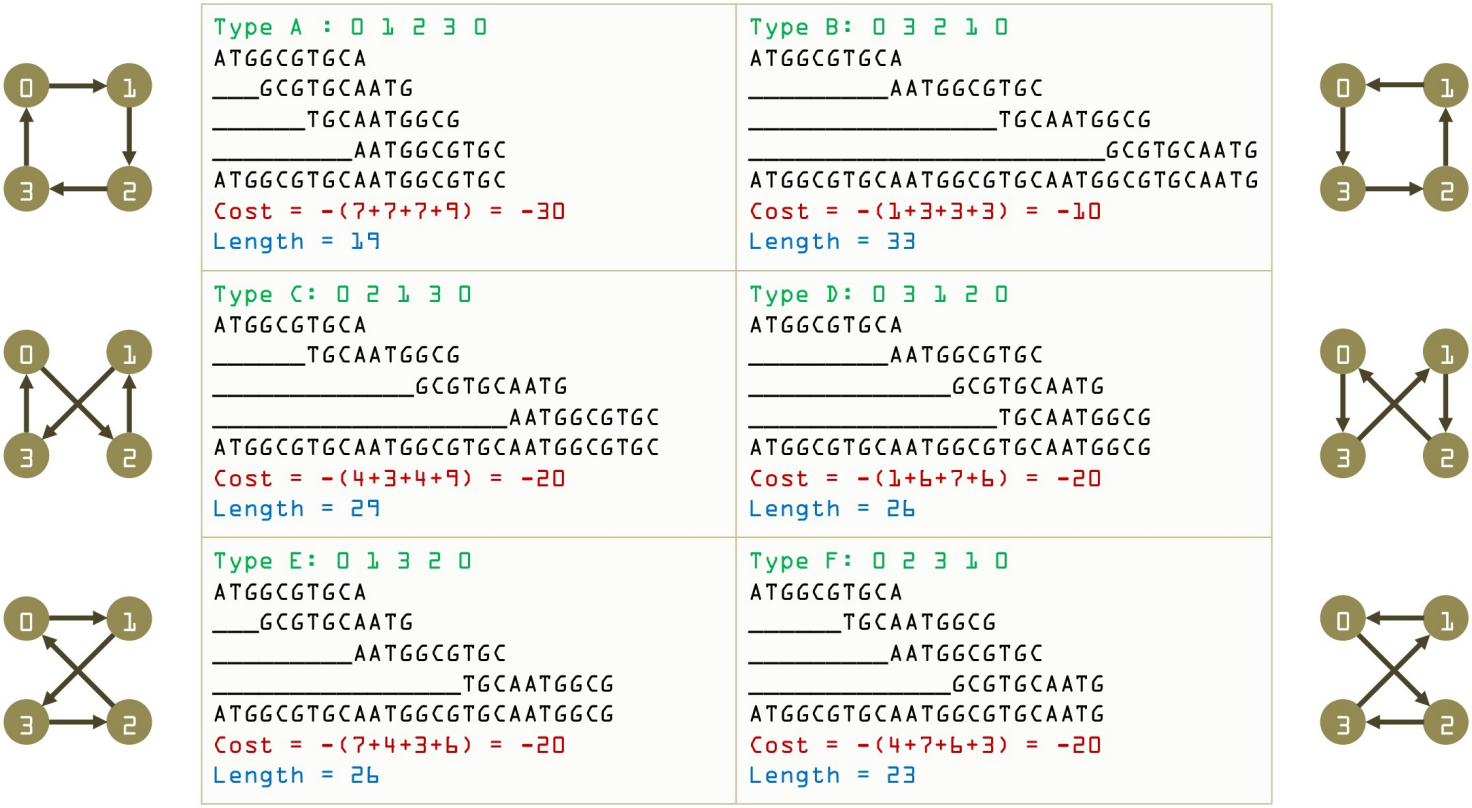
All possible TSP tours for given example.

The overlap depends on the ordering of the read pairs and thus this formulation is a directed graph. There are 6 possible unique tours (choosing a different starting city in the tour is equivalent in cost). Note that the reads are spliced from an original circular DNA, so the final stitched DNA solutions for all these tours are repeats of *read 0* of variable length. Such cases occur in practice when arranging the reads in different ways gives different repeat lengths. The tours are emulated in Fig 5. The TSP solution is expected to find the lowest cost (and shortest assembly) tour, i.e. Type-A. There are 4 acceptable solutions (based on starting node) of Type-A:

- (0 → 1 → 2 → 3 → 0)
- (1 → 2 → 3 → 0 → 1)
- (2 → 3 → 0 → 1 → 2)
- (3 → 0 → 1 → 2 → 3)

This process is automated in the classical pre-processing align and reads_to_tspAdjM functions in https://github.com/QE-Lab/QuASeR/blob/master/QA_DeNovoAsb/denovo_009.py. It can be invoked as

1 reads = [‘ATGGCGTGCA’, ‘GCGTGCAATG’, ‘TGCAATGGCG’, ‘AATGGCGTGC’]

2 tspAdjM = reads_to_tspAdjM (reads)

### TSP to QUBO model

Next, the directed TSP is encoded as a QUBO model. Let *n* = |*V*| = 4 be the number of nodes. This formulation [22] requires *n*^2^ = 16 binary variables as qubits, so it scales quadratically rather than linearly in the problem size. For *i*, *p* ∈ {0…(*n* − 1)}, let *x*_*i*,*p*_ be True if node *i* appears in position *p* in the cycle, False otherwise.

To derive a Hamiltonian for this problem, we penalize the violation of the constraints in the objective function inserting terms of the form $\alpha {\left(\sum_{p=0}^{n-1}x_{i,p}-1\right)}^{2}$, where the penalty term is sufficiently large, e.g., *α* = *n* * *max*_(*i*, *j*)∈*E*_ *w*_*ij*_. The TSP can be formulated as:

$$\begin{array}{c} \forall i,p\in \left\{0\ldots \left(n-1\right)\right\},x_{i,p}\in \left\{0,1\right\} \end{array}$$

**Fig 6:**
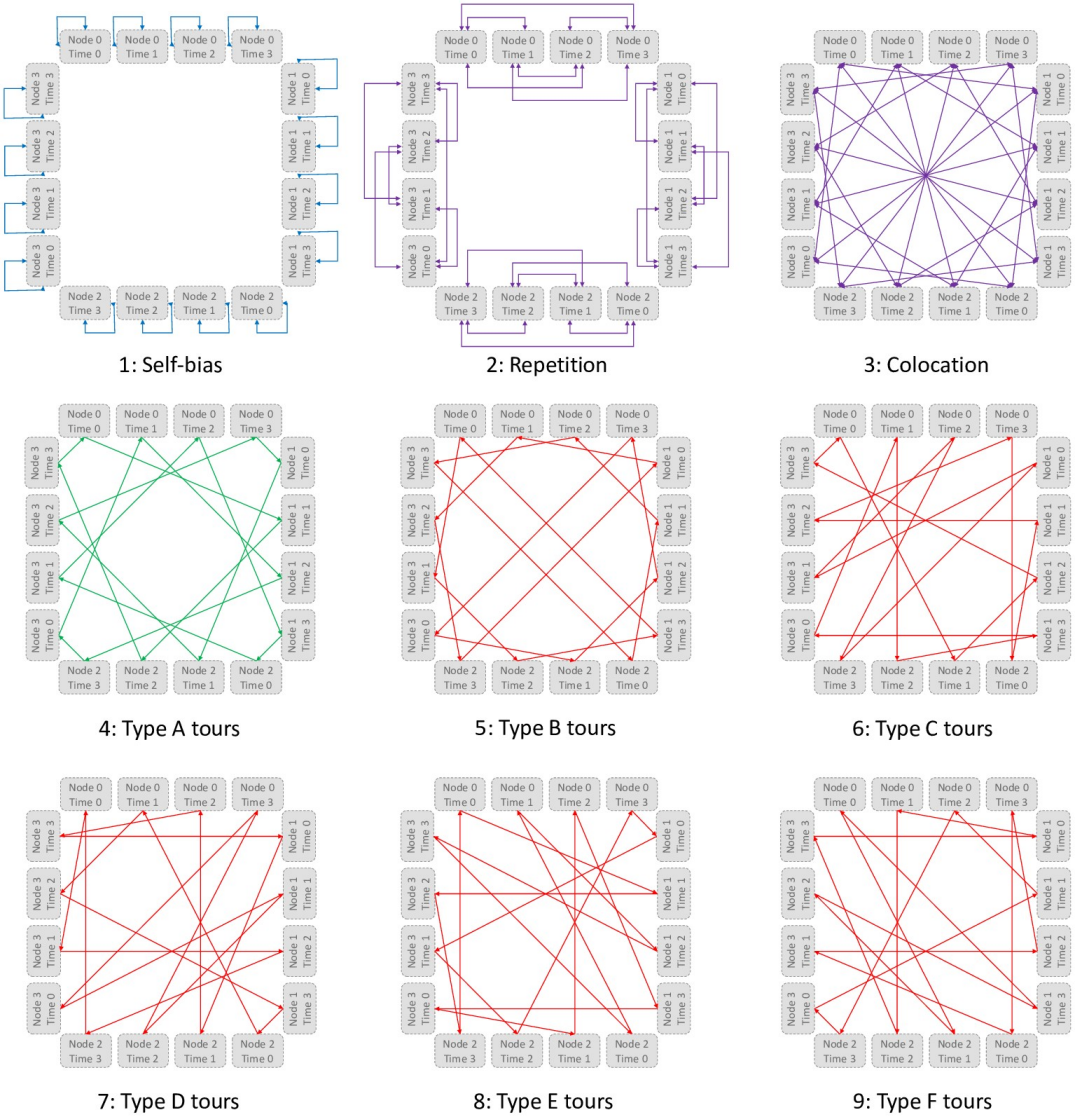
QUBO interactions.

The interactions are shown in Fig 6 and are categorized as:

1. Every node must be assigned. Thus self-interactions have large negative weight (favorable bias). Since there are no preferred order of the route, for each time slot, the value is the same (top-left blue interactions).
2. Same node assigned to two different time slots incurs a penalty (top-middle violet interactions). Thus, for each node, there should be only one assigned time slot:
  $$\begin{array}{c} \forall i\in \left\{0\ldots \left(n-1\right)\right\},\sum_{p=0}^{n-1}x_{i,p}=1 \end{array}$$
3. Same time slot assigned to two different nodes incurs a penalty (top-right violet interactions). Thus, for each time slot, there should be only one assigned node:
  $$\begin{array}{c} \forall p\in \left\{0\ldots \left(n-1\right)\right\},\sum_{i=0}^{n-1}x_{i,p}=1 \end{array}$$
4. The additional cost of including an edge in the route to two consecutive time slots is the weight of the edge in the TSP. These 6 graphs show the 4 possible routes for each of the 6 types (type A in middle-left green, others in red). Each edge (*i*, *j*) is taken and all possible configurations of assigning them next to each other are tried (the addition being modulo *n*), with the edge weight being the cost of choosing from those configurations. Thus, given the above constraints:
  $$\begin{array}{c} \text{minimize}:\;\sum_{i=0}^{n-1}\sum_{j=0}^{n-1}w_{ij}\sum_{p=0}^{n-1}x_{i,p}x_{j,p+1} \end{array}$$

**Fig 7:**
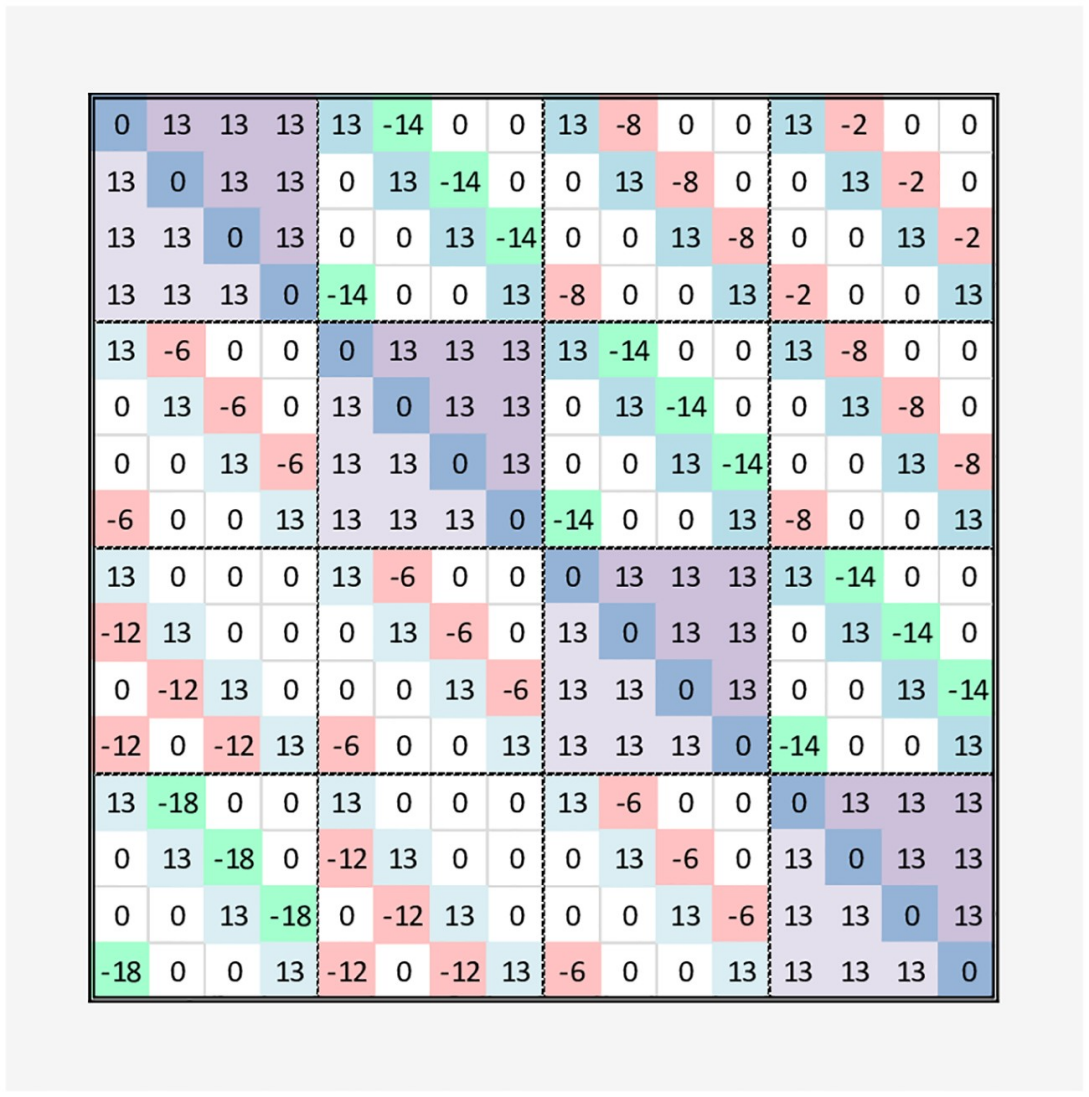
Q-matrix for de novo example.

These arrows can be made into an adjacency matrix for the graph, resulting in the Q-matrix as shown in Fig 7 (with the colors of the cells representing the terms from the corresponding colored arrows). The addition of these 6 matrices gives the *Q* matrix for the QUBO. Note that, if we have only the lower 6 matrices (coupling), the all 1’s assignment is the most favorable and gives the minimum solution to the QUBO equation *y* = *x*^*T*^ *Qx*. Thus, we need to add the reward for assigning a node {*a*} and penalties {*b*, *c*} for assigning the same node to multiple different time slots, or same time slots to multiple nodes, respectively. Since these are bi-directional arrows, these can be symmetric.

For our experiment, we empirically found that setting a penalty value of *b* = *c* ≥ 13 is sufficient, and the reward *a* = 0 still finds the 4 favorable minima of Type A. It is easy to verify that a minimum value of *y* = *x*^*T*^ *Qx* is obtained for:

- *x*^*T*^ = [1000010000100001]
- *x*^*T*^ = [0100001000011000]
- *x*^*T*^ = [0010000110000100]
- *x*^*T*^ = [0001100001000010]

for the binary encoding

$$\begin{array}{c} x^{T}=[n_{0}t_{0}|n_{0}t_{1}|n_{0}t_{2}|n_{0}t_{3}|n_{1}t_{0}|n_{1}t_{1}|n_{1}t_{2}|n_{1}t_{3}|n_{2}t_{0}|n_{2}t_{1}|n_{2}t_{2}|n_{2}t_{3}|n_{3}t_{0}|n_{3}t_{1}|n_{3}t_{2}\left|n_{3}t_{3}\right] \end{array}$$

The process of generating the Q matrix is automated in the function tspAdjM_to_quboAdjM in ‥/denovo_009.py. It can be invoked with the adjacency matrix from the reads_to_tspAdjM function as

1 #Parameters: adj matrix, self–bias, multi − location, repetation

2 quboAdjM = tspAdjM_to_quboAdjM (tspAdjM, 0, 13, 13)

## Solving on a quantum system

In this section, we show how to solve the QUBO on both a quantum annealer (D-Wave Ocean tools) and a gate-based optimizer (QX/OpenQL) using QAOA.

### QUBO using quantum annealing

The QUBO is mapped to a quantum annealer using the biases and couplings in the Ising model. A bias value is defined for each qubit and a coupling for each pair of qubits. In the graph view, each node (bias) and each edge (coupling) can have a real-number value (weight). Since this example requires 16 QUBO variables (qubits), the exact solver is used to better understand the output.

For the de novo example, the Q matrix from (with *a* = 0, *b* = *c* = 13) is shown in Fig 7.

The Q matrix is converted to a dictionary of node names and reward/penalty for biases and couplings. Then the QUBO solver is used to solve the Q matrix using the assignment of {0, 1} (instead of the Ising {−1, + 1}). This is coded in the quboAdjM_to_quboDict and solve_qubo_exact functions in ‥/denovo_009.py. It can be invoked with the qubo adjacency matrix from the tspAdjM_to_quboAdjM function as

1 Q = quboAdjM_to_quboDict (Q_matrix)

2 solve_qubo_exact (Q)

We find that there are 4 minimum solutions (the 4 Type A solutions). This matches with the expected analytical results.

Though the QUBO solution now works on D-Wave’s solver, it needs to be converted to the Ising model for it to run on the D-Wave Quantum Annealer. This can be done using the qubo_to_ising function in D-Wave’s toolset, which maps the definitions of binary variables to an Ising model defined on spins (variables with -1, +1 values). The following script solves the Ising model for our formulation.

Mathematically, the transform is: *x*^*T*^ *Qx* = offset + *s*^*T*^ *Js* + *h*^*T*^ *s*. For every linear (diagonal) bias term in Q, *h*[*i*] + = 0.5 * *Q*[*i*][*i*], while for each couplings, *J*[(*i*, *j*)] = 0.25 * *Q*[*i*][*j*], *h*[*i*] + = 0.25 * *Q*[*i*][*j*], *h*[*j*] + = 0.25 * *Q*[*i*][*i*]. The offset value is the weighted sum of the linear offset (sum of all diagonal terms in Q), and the quadratic offset (sum of all off-diagonal terms in Q), with the weights as 0.5 and 0.25, respectively. The offset is not important for our case as we want the qubit state of the minimum energy, not the exact value of the minimized energy. This is coded in the solve_ising_exact function in ‥/denovo_009.py. It can be invoked with the output of dimod.qubo_to_ising function as

1 hii, Jij, offset = dimod.qubo_to_ising (Q)

2 solve_ising_exact (hii, Jij)

As expected, we find that there are 4 minimum solutions (the 4 Type A solutions).

{‘n0t0’: +1, ‘n0t1’: −1, ‘n0t2’: −1, ‘n0t3’: −1,

‘n1t0’: −1, ‘n1t1’: +1, ‘n1t2’: −1, ‘n1t3’: −1,

‘n2t0’: −1, ‘n2t1’: −1, ‘n2t2’: +1, ‘n2t3’: −1,

‘n3t0’: −1, ‘n3t1’: −1, ‘n3t2’: −1, ‘n3t3’: +1}

{‘n0t0’: −1, ‘n0t1’: +1, ‘n0t2’: −1, ‘n0t3’: −1,

‘n1t0’: −1, ‘n1t1’: −1, ‘n1t2’: +1, ‘n1t3’: −1,

‘n2t0’: −1, ‘n2t1’: −1, ‘n2t2’: −1, ‘n2t3’: +1,

‘n3t0’: +1, ‘n3t1’: −1, ‘n3t2’: −1, ‘n3t3’: −1}

{‘n0t0’: −1, ‘n0t1’: −1, ‘n0t2’: +1, ‘n0t3’: −1,

‘n1t0’: −1, ‘n1t1’: −1, ‘n1t2’: −1, ‘n1t3’: +1,

‘n2t0’: +1, ‘n2t1’: −1, ‘n2t2’: −1, ‘n2t3’: −1,

‘n3t0’: −1, ‘n3t1’: +1, ‘n3t2’: −1, ‘n3t3’: −1}

{‘n0t0’: −1, ‘n0t1’: −1, ‘n0t2’: −1, ‘n0t3’: +1,

‘n1t0’: +1, ‘n1t1’: −1, ‘n1t2’: −1, ‘n1t3’: −1,

‘n2t0’: −1, ‘n2t1’: +1, ‘n2t2’: −1, ‘n2t3’: −1,

‘n3t0’: −1, ‘n3t1’: −1, ‘n3t2’: +1, ‘n3t3’: −1}

#### Using D-Wave quantum annealer

D-Wave offers a connection to the cloud to solve an Ising model. The problem needs to be embedded in the connectivity graph of the annealer. It is called a Chimera graph for the D-Wave 2000Q systems. Each of the 16 logical qubits is embedded over multiple qubits on the actual hardware so that each qubit shares a coupling based on the required interaction for the Ising model. The embedding process is a hard problem in itself and heuristics are employed in the D-Wave’s embedding function. Thus, with each run, the number of qubits and the longest chain length (i.e. the number of physical qubits encoding a single qubit) might vary, and even fail at times. The embedding process can be separately tested.

This is coded in the embed_qubo_chimera function in ‥/denovo_009.py. It can be invoked with the output of dimod.qubo_to_ising function as

1 embed_qubo_chimera (quboAdjM)

**Fig 8:**
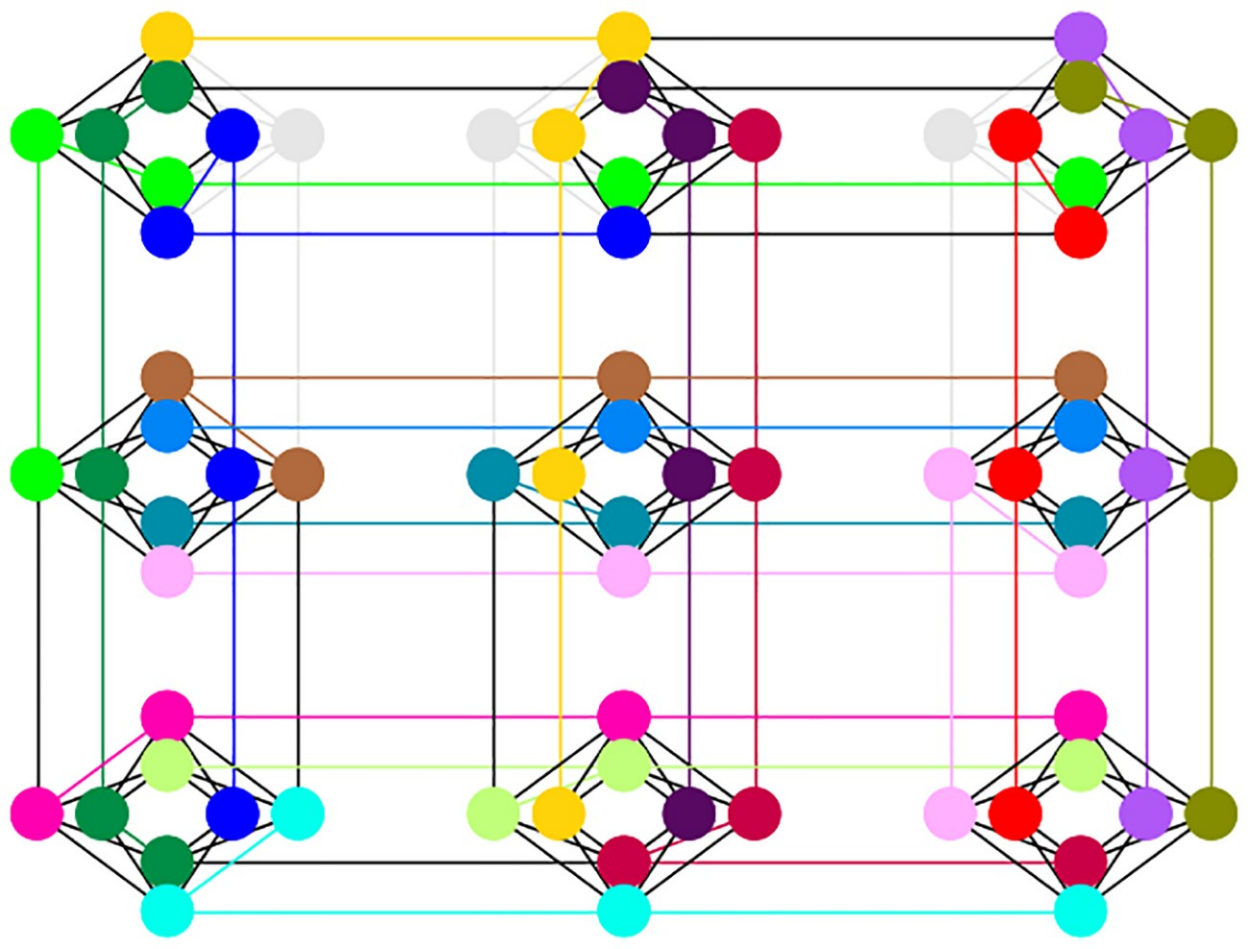
Embedding the QUBO in Chimera graph topology for the D-Wave quantum annealer. Each color represents one of the 16 logical qubits.

After multiple attempts, the best embedding obtained for our example de novo problem uses 60 qubits with a maximum chain length of 5, as shown in Fig 8. Each color represents one of the 16 logical qubits.

The following code connects to the D-Wave cloud and solves the de novo example. The code to connects to the D-Wave cloud and solve the de novo is in the solve_ising_dwave function in ‥/denovo_009.py. It can be invoked with the biases and coupling output of the dimod.qubo_to_ising function as

1 solve_ising_dwave (hii, Jij)

The top 10 (out of 65536) maximum sampled configurations are shown below. It is important to note that, the highest sampled configuration is not the global minima in terms of energy, showing the heuristic nature of the annealer.

Maximum Sampled Configurations from D−Wave ⇒

([−1,−1,−1,+1,−1,−1,−1,−1,+1,−1,−1,−1,−1,−1,+1,−1], −27.92288250, 4562)

([−1,−1,+1,+1,−1,−1,−1,−1,+1,−1,−1,−1,+1,−1,−1,−1], −22.70124548, 611)

([−1,−1,+1,+1,−1,−1,−1,−1,−1,+1,−1,−1,+1,−1,−1,−1], −26.16476862, 481)

([−1,−1,+1,+1,−1,−1,−1,−1,+1,−1,−1,−1,+1,−1,−1,−1], −22.70124548, 474)

([−1,−1,−1,+1,−1,−1,−1,−1,−1,+1,−1,−1,−1,−1,+1,−1], −28.08099638, 470)

([−1,−1,−1,+1,−1,−1,−1,−1,+1,−1,−1,−1,−1,−1,+1,−1], −27.92288250, 343)

([+1,−1,−1,−1,−1,−1,−1,−1,−1,+1,−1,−1,−1,−1,+1,−1], −27.81747324, 295)

([−1,−1,−1,+1,−1,−1,−1,−1,+1,−1,−1,−1,−1,−1,+1,−1], −27.92288250, 259)

([−1,−1,−1,+1,−1,−1,−1,−1,−1,−1,+1,−1,+1,−1,−1,−1], −27.60665473, 200)

([−1,−1,−1,+1,−1,−1,−1,−1,−1,−1,+1,−1,+1,−1,−1,−1], −27.60665473, 187)

The top 10 (out of 65536) minimum energy configurations are shown below. The list shows that, though the D−Wave was able to sample two of the four correct solutions, it has not sampled it with a high probability. Also, we find two other solution configurations are missed. Each run of the sampler would be slightly different varying both on environmental errors of the physical qubit system as well as the heuristics of embedding and schedule. Thus, while we were able to find an acceptable solution by the physical system, it might not be practical for larger problems.

Minimum Energy Configurations from D−Wave ⇒

([−1,−1,+1,−1,−1,−1,−1,+1,+1,−1,−1,−1,−1,+1,−1,−1], −30.41886117, 26)

([−1,−1,+1,−1,−1,−1,−1,+1,+1,−1,−1,−1,−1,+1,−1,−1], −30.41886117, 2)

([−1,−1,+1,−1,−1,−1,−1,+1,+1,−1,−1,−1,−1,+1,−1,−1], −30.41886117, 2)

([−1,−1,−1,+1,+1,−1,−1,−1,−1,+1,−1,−1,−1,−1,+1,−1], −30.41886117, 29)

([−1,−1,−1,+1,+1,−1,−1,−1,−1,+1,−1,−1,−1,−1,+1,−1], −30.41886117, 1)

([−1,−1,−1,+1,+1,−1,−1,−1,−1,+1,−1,−1,−1,−1,+1,−1], −30.41886117, 7)

([−1,−1,+1,−1,−1,−1,−1,+1,+1,−1,−1,−1,−1,+1,−1,−1], −30.41886117, 1)

([−1,−1,−1,+1,−1,+1,−1,−1,+1,−1,−1,−1,−1,−1,+1,−1], −29.89181489, 23)

([+1,−1,−1,−1,−1,−1,+1,−1,−1,−1,−1,+1,−1,+1,−1,−1], −29.89181489, 5)

([−1,−1,−1,+1,−1,+1,−1,−1,+1,−1,−1,−1,−1,−1,+1,−1], −29.89181489, 3)

A 16 qubit system was required for solving the above problem. When mapping the 16 qubits to a realistic hardware like D-Wave 2000Q, the connectivity of the qubits in the physical topology is important. The embedding process considerably increases the number of required qubits and also the quality of the solution. The highest number of DNA reads that can be solved on a D-Wave 2000Q machine is 9. The amount of qubits needed to solve the problem grows as *N*^2^ and finding embedding for the case with 10 reads will fail in most (if not all) cases. However, the time to solution is independent of the problem size and depends on a heuristic annealing schedule while affecting the quality of the sampled solution. In classical computation however, the record for exact solutions to the problem, using branch and bound algorithms is 85900 cities TSP [23]. Heuristics like Monte Carlo methods are used for larger inputs. This experiment infers the need for a much enhanced D-Wave system to do practical de novo assembly. Steps in this direction can be either in reducing the errors, having a custom anneal schedule, having more qubits and better connectivity (like the Pegasus architecture of the 5000 qubit model). At the current state of development, we were able to show a simple proof of concept both on the simulator and the quantum annealer on the cloud. The implementation is focused on the correctness of the pipeline design instead of quantifying the time and qubit resource metrics, that need radical improvements for benchmarking with real world datasets. De novo sequencing on quantum annealers needs to be evaluated with each release of improved hardware to reach a quantum advantage in computation over existing high-performance computing systems.

### QUBO using QAOA

Formulating a problem on QAOA involves specifying the ansatz for the cost and driver Hamiltonian. Other optimization hyper-parameters involve the initialization circuit, approximation order (cycles), initial parameters and threshold on classical optimizer iterations/precision. The pseudo-code for the formulation steps in an OpenQL implementation is shown in Box 2.

**Box 2. Pseudo-code for applying QAOA in OpenQL for optimization.**

• PQC encoding (e.g. Traveling salesman problem, Maximum cut problem):

 ◊ Create (weighted) graph with networkx

 ◊ Converts graph to weighted-Sum-of-Product-of-Paulis (wsopp). Encoding depends on problem. This is the problem/cost Hamiltonian for QUBO/Ising model.

 ◊ Convert graph to ansatz with cost and mixing Hamiltonian as parameterized QASM (ansatz, coefficients, angle ids)

• Initialization:

 ◊ Classical optimizer object from SciPy:

  ⋆ name [Default: Nelder-Mead]

  ⋆ convergence function tolerance [Default: 1.0e–6]

  ⋆ iteration limit

 ◊ Reference/initial state quantum circuit [Default: equal superposition, Hadamard on all qubits]

 ◊ Steps (ansatz blocks per iteration) [Default: 1]

 ◊ Parameters:

  ⋆ for cost Hamiltonians (gammas) for each step

  ⋆ for mixing/driving Hamiltonians (betas) for each step

  [Default: random angles in 0 to 2*π*]

 ◊ Shots (for state tomography measurement aggregate) [Default: 0, QX internal state vector is accessed]

• Invoke QAOA

First, the TSP city-graph is created based on the previous example. The networkx Python package is used to create a directed weighted complete graph based on the pair-wise read edge-weights.

The graph is then converted to the problem Hamiltonian. The problem Hamiltonian is stored as a weighted sum-of-product of Paulis. For *n* cities (TSP graph nodes), *n*^2^ qubits are required representing the city nodes and the time slots, encoded as:

$$\begin{array}{c} q0\ldots q15\equiv [n_{0}t_{0}|n_{0}t_{1}|n_{0}t_{2}|n_{0}t_{3}|n_{1}t_{0}|n_{1}t_{1}|\ldots |n_{3}t_{2}|n_{3}t_{3}] \end{array}$$

On each qubit, there can be either of the 4 Pauli operators, {*I*, *X*, *Y*, *Z*}, thus a maximum of $4^{n^{2}}$ weighted sum-of-product Pauli terms are possible. This amounts to 4294967296 Pauli terms when *n* = 4 (in our example), thus, we store only the non-zero terms. For TSP optimization, however, only the {*I*, *Z*} operator is required.

Firstly, each city and each time-slot must be assigned, but not all together. Thus, a term is added with a positive penalty (*w* = 100000.0) for each qubit (the term being a *Z* operator on the specific qubit and *I* otherwise). We will abbreviate the sum-of-product of Pauli term notation henceforth by assuming Identity for qubits not mentioned in a Pauli term. Thus:

$$\begin{array}{c} w*\{ZIIIIIIIIIIIIIII+IZIIIIIIIIIIIIII+\ldots +IIIIIIIIIIIIIIIZ\} \end{array}$$

$$\begin{array}{c} H_{C}^{1}=w*\left\{Z_{0}+Z_{1}+Z_{2}+\ldots +Z_{15}\right\}=\sum_{q=0}^{n^{2}-1}wZ_{q} \end{array}$$

Then, for each penalty of co-location (two cities, same time slot), a term with 2 *Z* operators for the two conflicting qubits is added with a positive penalty weight, and two separate terms for each penalty qubits with a 1 *Z* are added with a negative penalty.

$$\begin{array}{c} H_{C}^{2}=\sum_{r=0}^{n-1}\sum_{i=1}^{n-1}\sum_{j=0}^{i-1}\{-\frac{w}{2}Z_{in+r}-\frac{w}{2}Z_{jn+r}+\frac{w}{2}Z_{in+r}Z_{jn+r}\} \end{array}$$

Similar terms are added for repetition (two time slots, same city).

$$\begin{array}{c} H_{C}^{3}=\sum_{i=0}^{n-1}\sum_{r=1}^{n-1}\sum_{s=0}^{r-1}\{-\frac{w}{2}Z_{in+r}-\frac{w}{2}Z_{in+s}+\frac{w}{2}Z_{in+r}Z_{in+s}\} \end{array}$$

For each TSP edge, the edge pair is assigned to consecutive time slots. The edge weight is added as a penalty for the 2 *Z* operator terms, while assigning the individual terms with a negative penalty.

$$\begin{array}{c} H_{C}^{4}=\sum_{i=0}^{n-1}\sum_{\begin{array}{c} j=0\\ j\neq i \end{array}}^{n-1}\sum_{\begin{array}{c} r=0\\ s=(r+1)\%n \end{array}}^{n-1}\{-\frac{d_{ij}}{4}Z_{in+r}-\frac{d_{ij}}{4}Z_{jn+s}+\frac{d_{ij}}{4}Z_{in+r}Z_{jn+s}\} \end{array}$$

Thus, these negative penalty terms in the overall equation stand out among the positive penalty terms for all qubits in $H_{C}^{1}$, so that only a valid path is assigned as a solution. The final cost Hamiltonian is $H_{C}=H_{C}^{1}+H_{C}^{2}+H_{C}^{3}+H_{C}^{4}$.

Now the ansatz needs to be formed. For the cost Hamiltonian ansatz, for each single *Z* terms, the Pauli-Z is replaced with a parameterized Z-Rotation gate *R*_*Z*_(*γ*). The double *Z* terms between qubits (*a*, *b*) are replaced with a $CNOT_{ab}R_{Z}^{b}\left(\gamma \right)CNOT_{ab}$ (where *b* is the target qubit). For the mixing Hamiltonian, a *R*_*X*_(*β*) is used on all qubit (or *HR*_*Z*_(*β*)*H*).

The reference state is set to an equal superposition (Hadamard over all qubits). The cost and mixing Hamiltonian ansatz is alternated for the set number of steps (1 in our case). This forms the parametric circuit for the quantum computer. Along with this, the random initial parameters vector (Γ, *B*) is passed to the classical optimizer wrapper for the QAOA. The QAOA is run as explained in Box 1.

**Fig 9:**
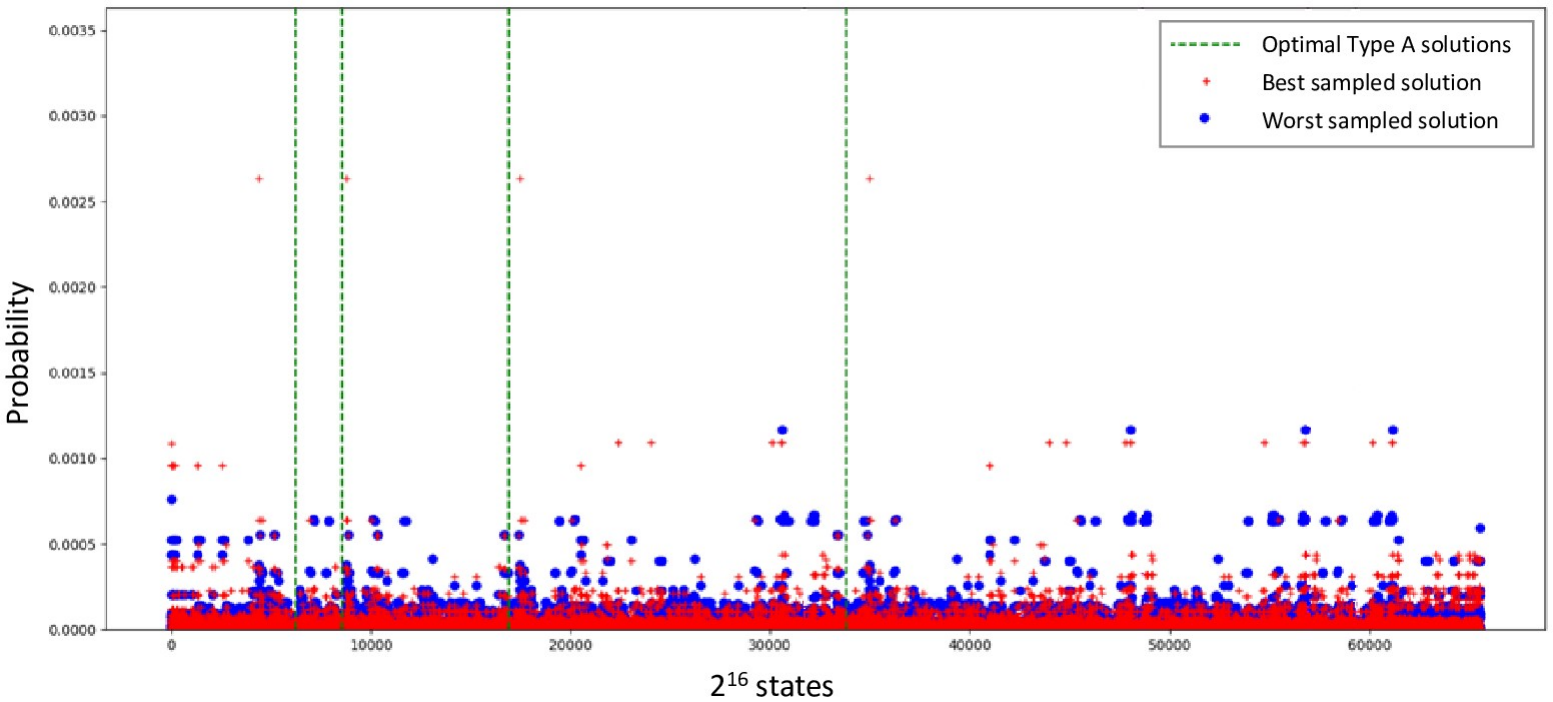
Result of simulating QuASeR example using QAOA on the QX simulator.

We simulated our algorithm on the QX Simulator to validate the results. To speed up the simulation, we access the internal state vector from the QX Simulator instead of applying the state tomographic trials. However, optimizing the parameters on 16 qubits for single iteration over a single QAOA step proved to be cumbersome, reaching the limit of the working memory for most runs. While we obtain good results for the Max-Cut problem (which requires as many qubits as nodes in the graph), the *n*^2^ qubit space for TSP is costly for simulation. Similarly, most online tools only offer examples for the trivial case of an undirected triangle graph (where only one Hamiltonian cycle is possible). We used the Nelder-Mead, Powell and BFGS optimizers in SciPy. We found that the optimizers are able to explore only a small space near the initial guess before settling at a suboptimal solution. This is shown in Fig 9, where the dotted vertical green lines indicate the 4 optimal type A solution states out of the 2^16^ = 65536 basis states (represented on the x-axis). The experiment uses a QAOA depth of 1, a random angle initialization, an equal superposed initial state (Hadamard on all qubits) and 40 reruns for the QAOA optimizer. When the initial guess is bad, the highest probability states (high blue circles) are not close to the optimal lines, whereas for good initial conditions case (red pluses), the optimizer eventually reaches solutions that are quite close to the optimal. The optimal solutions lie in near vicinity of the found solutions (high red pluses) and can be subsequently explored via an exhaustive search near these sub-optima suggested by QAOA. QAOA is able to improve 2 out of the 4 solutions of Type A. Development of new classical optimizers [24, 25] and their hyper-parameter settings [26] for HQC algorithms is a research field of its own, which is not the focus of this paper. Thus, while we were able to formulate a generic de novo assembly problem to QAOA, we were not able to obtain satisfactory results from the simulation. This motivates the need for both faster simulation and the access to better NISQ devices where this entire pipeline can be executed and benchmarked—a common challenge for quantum computing today.

QAOA, though promising for exhibiting “quantum supremacy” does not imply that it will be able to outperform classical algorithms on important combinatorial optimization problems such as Constraint Satisfaction Problems. Current implementations of QAOA are subject to a gate fidelity limitation, where the potential advantages of larger values of the parameter *p* in QAOA applications are likely to be countered by a decrease in solution accuracy.

## Conclusion

In this paper, we formulated a de novo assembly DNA sequence reconstruction algorithm called QuASeR using the overlap-layout-consensus approach for acceleration on a quantum computing platform. The quantum kernel is formulated for both a gate-based quantum system as well as a quantum annealer. The required technical background for formulating the de novo sequencing problem (i.e. QUBO, TSP, and Hamiltonians) is introduced with simple examples to target both the genomics research community and quantum application developers. A proof-of-concept de novo sequence assembly is mapped to TSP and then to a QUBO. This is firstly solved on the D-Wave simulator and D-Wave Quantum Annealer. All 4 correct results are obtained on the simulator while only 2 of the solutions are sampled on the Quantum Annealer (though with less probability). The connectivity topology of the D-Wave architecture limits embedding larger problem instances. The variational algorithm approach for gate-based quantum computing is introduced for solving optimization problems using QAOA. This algorithm performs two optimization steps, one executed on a quantum circuit and another on a classical computer. The proposed de novo algorithm is solved using QAOA, and then simulated on the QX simulator. Simulation showed that the results are heavily dependent on the exploratory capabilities of the classical optimizer. A gate-based quantum computer is not targeted as the coherance time, connectivity topology and number of qubits prevents any meaningful result at the current state of available quantum hardware. This research is part of a full-stack domain-specific quantum accelerator project [6] undertaken in the Quantum Computer Architecture lab at the Delft University of Technology. It is the first time this important computation problem in bioinformatics is attempted on a quantum accelerator.
